# Women’s experiences of being invited to participate in a case-control study of stillbirth - findings from the Midlands and North of England Stillbirth Study

**DOI:** 10.1186/s12884-018-1956-1

**Published:** 2018-08-06

**Authors:** Jayne Budd, Tomasina Stacey, Bill Martin, Devender Roberts, Alexander E. P. Heazell

**Affiliations:** 1 Maternal and Fetal Health Research Centre, School of Medical Sciences, Faculty of Biological, Medical and Human Sciences, University of Manchester, Manchester, UK; 2 Manchester Academic Health Science Centre, St. Mary’s Hospital, Central Manchester University Hospitals NHS Foundation Trust, Manchester, UK; 3 School of Healthcare, University of Leeds, Leeds, UK; 4 Birmingham Women’s Hospital NHS Foundation Trust, Mindelsohn Way, Edgbaston, Birmingham, UK; 5 Liverpool Women’s Hospital NHS Foundation Trust, Crown Street, Liverpool, UK

**Keywords:** Stillbirth, Research participation, Research recruitment

## Abstract

**Background:**

The Midlands and North of England Stillbirth Study (MiNESS) was a case-control study of women who had a stillbirth or who had an ongoing pregnancy. During the set up phase questions were raised about whether interviewing women within six weeks of a stillbirth and recruiting women who were still pregnant into a “stillbirth” study was acceptable. This led to the research questions “whether it is appropriate to ask women who have recently experienced a stillbirth to participate in research?” and “whether it is appropriate to ask pregnant women to participate in a research project looking at factors associated with stillbirth.” This nested study aimed to describe the opinions of women approached to participate in MiNESS to explore their views and experiences of a research project focussed on stillbirth.

**Methods:**

Semi- structured interviews were conducted at a single study site involved in MiNESS. Purposive sampling was used to obtain a sample of women who were approached following a stillbirth (case *n* = 6) and those who were approached during pregnancy who gave birth to a live born baby (control *n* = 6). These two groups of women were divided equally according to whether they participated in the main MiNESS questionnaire study and those who declined to do so (*n* = 3 in each group). Interview data were transcribed and analysed using thematic analysis to identify the most important factors in determining whether women participated in MiNESS.

**Results:**

The following themes emerged from the analysis: participants’ understanding of research; approach by researcher; wanting to help; stillbirth taboo. These themes are explored individually in the manuscript. Participants reported positive views about research and previous participation in research studies. Respondents valued an initial approach from a member of staff already known to them. The taboo around stillbirth was a barrier to participation for some women with ongoing pregnancies.

**Conclusions:**

Experiences and views regarding research differed between participants and non-participants in the MiNESS study. Participants reported a greater understanding of the importance and implications of clinical research. When designing future studies, the timing of approach, clarity of information and the person approaching potential participants should be considered to optimise recruitment.

**Trial registration:**

NCT02025530 date registered: 01/01/2014.

## Background

The UK has one of the highest rates of stillbirth in high-income countries, with more than 3000 stillbirths every year [1]. The rate of stillbirth has only decreased at 1.4% per year between 2000 and 2015 over the last 20 years [2], which urgently needs to be addressed to achieve the UK Government’s targeted reduction by 50% by 2025 [3]. Yet, in comparison to other pregnancy complications stillbirth is under-researched, for example using “stillbirth” as a search term in PubMed yields 8897 articles, compared to 38,066 for “preeclampsia” and 25,811 for “preterm labour” (Searches completed 7th March 2018). This paucity of evidence has been highlighted in several systematic reviews [4, 5]. Prior to commencing our stillbirth research programme we conducted a research priority setting partnership in collaboration with the James Lind Alliance [6], one third of responses to this exercise were from parents. This identified 11 priorities for stillbirth research, most of these required clinical studies to answer them; while some studies only needed to recruit bereaved parents others needed to recruit parents who had ongoing pregnancies and others with a history of stillbirth. Thus, progress in stillbirth research is dependent upon an ability to recruit participants who have experienced a stillbirth.

Few studies have reported participants’ views and experiences of studies relating to stillbirth. The Auckland Stillbirth Study [7], a case control study, recruited women who had recently experienced stillbirth and healthy pregnant women. All participants were interviewed face to face and an in depth questionnaire was completed. This study had high participation rates (72%), these were attributed to the willingness of recently bereaved women and currently pregnant women to participate. The investigators stated that they received no negative feedback, with pregnant women commenting that they wanted to help others whilst the women who had experienced stillbirth also expressed the desire to help others along with wanting to find answers and the opportunity to talk and share their experience [8]. Breeze et al. conducted a study investigating parental views about research participation around perinatal post-mortem which found that 73% of the participants felt better about the decision they had made about post mortem by completing the study questionnaire [9].

The Midland and North of England Stillbirth Study (MiNESS), a case-control study of women who had experienced a stillbirth (cases) and women who had an ongoing pregnancy ending in a live birth (controls) [10] recruited from April 2014 to March 2016. During the initial set-up phase questions were raised by research and development departments, consultant obstetricians, research midwives and bereavement midwives about interviewing women within six weeks of a stillbirth event and recruiting women who were still pregnant into a “stillbirth” study. This led to the development of the research questions “Is it appropriate to ask women who have very recently experienced a stillbirth to participant in research?” and “Is it appropriate to ask pregnant women to participate in a research project looking at factors associated with stillbirth?” To address these questions this nested study aimed to ask the opinions of women approached to participate in MiNESS to describe their views and experiences of a research project focussed on stillbirth. It was anticipated that this information would optimise recruitment strategies for use in future stillbirth research based on both the views of women who have experienced a stillbirth and healthy pregnant women.

## Methods

**Fig. 1:**
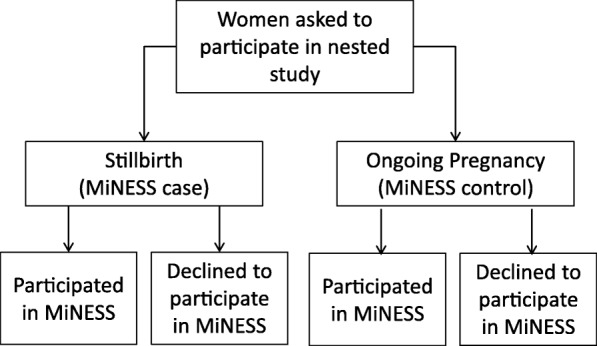
Schematic representation of how participants were grouped in this nested case-control study

This nested qualitative study was conducted in the largest study site involved in MiNESS – St Mary’s Hospital, Manchester, a tertiary maternity unit in the North-West of England. A favourable ethical opinion for the study was given by the Greater Manchester Central Research Ethics Committee (Ref 13/NW/0874). Purposive sampling was used to obtain a sample of women who were approached following a stillbirth (MiNESS case) and those who were approached during pregnancy (MiNESS control), within these two groups women were approached to participate in this sub-study who participated in the main MiNESS questionnaire study and those who declined to do so (Fig. 1). The overall sample size was determined prior to the interviews, based on Guest et al. [11] who suggested data saturation (when no new themes emerge) occurs around 12 participants. After six interviews had been conducted data saturation was reviewed iteratively after each interview had been transcribed and analysed to determine whether new themes had emerged.

Following a review of possible research methods, a semi-structured interview over the telephone was felt to be the most appropriate way to explore women’s views and experiences. However, as potential participants had already declined to participate in the original research project, ethical approval was not given for further contact, of these women as potential research participants have the right to decline to participate without having to give an explanation [12]. Therefore, women who consented to participate in MiNESS (cases and controls) were asked at the end of the study interview if they were happy to be contacted to participate in an additional interview. If they accepted, a participant information sheet was given and a suitable date and time was arranged to complete the interview. Women who declined to participate in MiNESS from both and case and control groups were sent a covering letter and participant information sheet and asked to return the reply slip if they were happy to be contacted about the additional study. If they agreed, a suitable date and time was arranged to complete the interview.

### Data collection

Participants were interviewed over the telephone by the research midwife at a date and time convenient to the participant. Telephone interviews were chosen for pragmatic reasons and following guidance from the ethics committee, as it was considered that face to face interviews would be impractical for women who had declined to participate in the main MiNESS study. Verbal consent was obtained for the interview to take place and for the interview to be audio recorded. The interview was transcribed verbatim. Women were recruited to this sub-study between June and July 2015; transcription was completed by December 2015. Analysis of the data was carried out between January to July 2017 after the main study had completed to avoid bias.

Themes are patterns across data sets that are important to the description of a phenomenon and are associated to a specific research question. Data obtained were analysed using thematic analysis according to the approach of Braun and Clarke. Thematic analysis is the most commonly used form of analysis in qualitative research [13]. The approach of Braun and Clarke emphasizes pinpointing, examining and recording patterns (or “themes”) within data. The themes become the categories for analysis. Applying Braun and Clarke’s method thematic analysis is performed through the process of coding in six phases to create established, meaningful patterns. These phases are: familiarization with data, generating initial codes, searching for themes among codes, reviewing themes, defining and naming themes, and producing the final report [13]. The coding strategy and arising themes were discussed with a co-author [AH].

## Results

A total of 12 women were recruited, 6 women who had a stillbirth and 6 women who were interviewed during pregnancy (all of whom went on to have a live birth). Each of these smaller groups contained three women who participated in the main MINESS questionnaire and three women who did not. This sample size represents 18% of all women approached to participate in the study at this centre. The characteristics of participants in this sub-study are shown in Table 1.

**Table 1:** Demographic characteristics of participants indicating whether participants in this study took part in MiNESS, and if so whether they were a case (a woman who had a stillbirth) or a control (a woman interviewed with a live ongoing pregnancy)

Participant in MiNESS	Group	Gestation at time of interview/ stillbirth diagnosis (weeks)	Gravidity / Parity prior to stillbirth/ birth	Age	Ethnicity
Yes	Case	31	G1 P0	25–30	White British
No	Case	34	G4 P1	25–30	White British
Yes	Case	41	G1 P0	20–25	White British
No	Control	40	G1 P0	30–35	Black Caribbean
Yes	Control	36	G2 P1	35–40	Black African
Yes	Case	40	G2 P0	30–35	White British
No	Case	41	G2 P1	25–30	Pakistani
No	Control	32	G1 P0	35–40	White British
Yes	Control	32	G3 P1	20–25	Bangladeshi
No	Control	28	G10 P3	25–30	White British
No	Case	38	G1 P0	35–40	Other
Yes	Control	33	G2 P1	25–30	White British

The following themes emerged from the analysis: participants’ understanding of research; approach by researcher; wanting to help; stillbirth taboo. These will be explored individually.

### Understanding of research

When asked about their understanding of clinical research the majority of those interviewed felt that they had a poor understanding of what research involved, the majority of respondents equated clinical research with drug trials or tests. In particular, some participants made negative comments about the (lack of) value of a non-interventional trial such as a questionnaire study.

*“I don’t really know. Suppose you mean drugs, err testing drugs?” (Case participant).*

*“Not sure, maybe tests, pills, but then you said this was research and you’re not testing anything are you? So that can’t be right it must be… erm… I don’t know” (Control - non- participant).*

*“It’s a pain, people stop you in the street, can you fill out a questionnaire, it’s just people being nosey” (control non- participant).*

*“I can’t see the point. I can see testing medicine but not just asking questions, what’s done’s done.” (Case - non-participant).*

In contrast, other participants had a clear understanding of research and viewed it positively, all these views were from women who participated in MiNESS. This suggests that women who have a better understanding of research may be more likely to participate.

*“I know its investigating things, anything, so you get medical research which tests medicines, care, thoughts on things like you’re doing. But you can also get market research, you know when your shopping or online, they ask things like what newspaper do you read- like anyone reads newspapers anymore (laugh)” (Case - participant).*

*“Research is good because it moves things forward, people don’t realise that without people doing research we wouldn’t have a lot of things. I mean things like how we’re cared for by the midwives and doctors. They only know how to do it because someone did research” (Control - participant).*

*“Everything we know is because of research I mean penicillin, who was the guy who found that? Erm… I can’t remember but you know what I mean” (Case - participant).*

### Approach by researcher

The approach methods for MiNESS were different for cases and controls. Cases were initially approached by a bereavement midwife whereas the controls were contacted directly by the researchers. This difference in approach may have had an effect on participation as potential participants reported different experiences about the approach to participate in research.

*“I was given the information before leaving hospital and was asked if <research midwives name> could call me, I just said yes. Then <bereavement midwives name> mentioned it again to me a couple of days later. Then I think <research midwives name> rang me and had a chat about the study it sounded good. The only thing I was a bit worried about was <research midwives name> coming to my house, I didn’t know her but she was lovely and <bereavement midwives name> had said she was before” (Case - participant).*

*“I don’t know really I think <research midwives name> rang me out of the blue, but she was nice on the phone so I said yes. I don’t think I really knew why she was ringing me but she sent me the information and then rang back again before coming to my house to see me” (Control - participant).*

*“It was find <bereavement midwives name> gave me the info and said <research midwives name> would ring so I was expecting her call” (Case - participant).*

*“I don’t think it was the best way, you know, I don’t even know the midwife, my own midwife should have mentioned it me then I would have been prepared for her calling I thought something was wrong saying she was from St Marys” (Control - participant).*

Although potential participants identified negative aspects regarding the approach to participant they also struggled to suggest alternative methods as they felt there was no easy way to introduce researchers, particularly when they were unfamiliar.

*“I was a bit scared as I didn’t know <research midwives name> and hadn’t met her perhaps if I’d have met her before leaving the hospital I would have felt better” (Case - participant).*

*“I think the lady rang me I didn’t know anything about it before I thought she was ringing for PPI (Payment Protection Insurance) or something (laughs) but then I was worried when she said she was ringing from St Mary’s as I thought something was wrong.”*

When asked to suggest an alternative approach this participant answered *“I don’t know a letter or something might have been better but then maybe I’d have thought it was junk mail. I don’t know” (Control - participant).*

Timing of the contact was another factor which women reported to influence participation. In particular for cases this reflected the timing of approach relative to the death of their baby, with one non-participant suggesting that a greater timeframe may have facilitated their participation.

*“It was too soon, I can remember thinking bloody hell let me get home and my head round this before you want to come and find out what’s happened.” (Case - non-participant).*

*“I didn’t know what day it was so couldn’t think about anything other that what was happening. Looking back if <research midwives name> had rung me after a month or so I would have said yes” (Case - non-participant).*

*“I think you called me at the wrong time, I was at work and didn’t really have time to talk. You know like PPI I just said ‘no thanks’ without understanding why or what.” (Control - non-participant).*

### Wanting to help

Of those who participated in the main MiNESS questionnaire their main reason for doing so was to help irrespective of their status (case/control).

*“Because I wanted to help, it must be terrible for someone to lose a baby so if I can help then I’m more than happy to, besides it was only a questionnaire” (Control - participant).*

*“I wanted to help stop this happening to someone else, no one can imagine how bad it is. I couldn’t stop it but if I can help stop it in future then I’m happy to help in any way.” (Case - participant).*

*“Because I’ve done research before I just said yes. Then when I actually found about what and why I was more than happy to help. I mean how awful it is. It’s the worst thing ever.” (Control - participant).*

### Stillbirth taboo

One of the reasons for not participating was the term “stillbirth”. Some women are concerned about jinxing their pregnancy or were confused by the purpose of the study.

“*I said no because I thought it might be bad luck, I know it’s silly but I was frightened when they said a stillbirth study” (Control - non-participant).*

*“I didn’t understand as I haven’t had a stillbirth so didn’t know why the midwife was calling me, she did explain but I was confused.” (Control - non-participant).*

Women who participated in MiNESS reported that they were initially concerned but consented after a full explanation, even then participants reported some anxieties about participating in a study on stillbirth.

*“Even though I said yes I was a bit worried about it before <research midwives name> came and talked to me face to face about it I then understood that it was nothing to do with stillbirth really.” (Control - participant).*

*“I didn’t know it was a stillbirth study until I read the information but by then I’d said yes so just carried on. It was ok actually <name of research midwife> was lovely and put my mind to rest.” (Control - participant).*

## Discussion

**Fig. 2:**
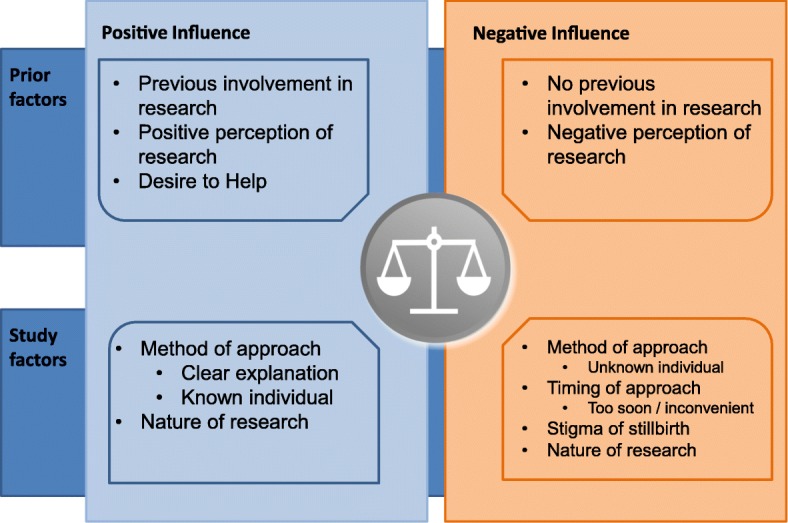
Schematic representation of themes which may exert a positive or negative influence on a woman’s willingness to participate in a research project relating to stillbirth

This nested qualitative study aimed to report the opinions of women approached to participate in the MiNESS questionnaire study to describe their experiences and views to inform future study design. The interviews provided rich information giving insight into why women participate or decline to participate in research studies focussing on stillbirth. Primarily, this study identified differing views and experiences of women who did and did not participate in the main MiNESS questionnaire study. This study identified themes particularly about the nature and timing of the approach to participate in a study about stillbirth which can be modified to optimise recruitment in future studies. The potential influence that the themes identified here could have on a woman’s decision to participate is shown in Fig. 2; these are divided into factors which exert a positive and negative effect in women’s willingness to participate in a study. Some themes, such as prior experience of research, could influence participation positively or negatively. For example, positive prior experience of research tended to promote participation due to positive views about the value of clinical research. Other themes, notably the taboo of stillbirth, only had a negative influence; whilst a desire to help only positively influenced participation. Importantly, the aim of reducing stillbirth was perceived as desirable by both cases and controls who participated.

This study described mixed thoughts and levels of understanding about clinical research which may influence participation, as women who participated in the main MiNESS questionnaire study were more likely to report an understanding of the importance and implications of clinical research. Conversely, women who did not participate had less understanding of the nature of research. Confusion or a lack of understanding about the specific study was also a barrier to participation, although this eased after discussion with the researcher in some cases but not always.

### Strengths and limitations

The interviews for this study were performed by researchers who had not been involved in MiNESS which enabled women to more freely decide whether to participate in the additional interview and speak honestly about their experiences. This qualitative study was also strengthened by inclusion of women from non-white British ethnic groups in similar proportion to women giving birth at the maternity unit. However, the participation rate in this nested study was low; to obtain consent from 12 participants 33 women were approached. Recruiting women who had experienced a stillbirth (cases) but declined the main MiNESS was particularly challenging and was usually achieved around 12 weeks after their baby had died. Due to the small numbers recruited in the study and recruitment from a single MiNESS study site, there were concerns that data saturation would not be achieved. However, no new themes emerged by the twelfth interview, therefore saturation was judged to be achieved. This is consistent with the findings of Guest et al. who [11] found that after conducting 60 in depth interviews, data saturation occurred within the first 12 interviews with elements of themes being present within the first six interviews. One reason for the comparatively low number of interviews to reach data saturation in this study is that themes were often mirrored between the two groups e.g. the role of staff members were either perceived positively or negatively.

This study may also be limited by the use of telephone interviews rather than face to face approach which allows additional observation of body language and participant behaviour. However, Novick [14] found that telephone interviews allowed participants to feel relaxed and able to disclose sensitive information with limited evidence demonstrating that they produce lower quality data.

The findings may not be transferable to other study sites or investigations using a different methodological approach. As research into stillbirth is limited these results are valuable as they give important insights into research participation for women who have experienced a stillbirth and those with ongoing pregnancies. Due to the small sample size it was not possible to analyse if there were differences in what was said by women of different ages or from different ethnic backgrounds, a larger sample size with purposive sampling of women from minority ethnic groups would be needed to complete this.

### Relevance of the study to patients, healthcare providers and researchers

This study has highlighted important themes from both women who have experienced stillbirth and pregnant women about the approach and participation in research projects and also identified specific issues relating to stillbirth research. This will inform future research studies and help to devise recruitment strategies which are amenable to women in future research projects into this sensitive area of maternity research.

In general there is a paucity of research looking at views and experiences of participating in research during pregnancy. Literature searches were performed to review available evidence; these reveal a small number of studies, most of which refer to either specific groups of participants of specific research topics. For example, Wendler et al. [15] looked at participation rates of different ethnic groups, and found very little differences in the willingness of potential participants from minority groups to participate in research and suggests the focus should be on ensuring access to research for all. Breeze et al. looked at parent’s attitudes to perinatal post-mortem following late miscarriage, stillbirth and termination for abnormality [9]. This found no participants reported any adverse effect of completing the research questionnaire and a majority of respondents (73%) reported feeling more secure in their decision (to have/not have a post-mortem) following their participation in the research project. Breeze et al. [9] identified three themes which were similar to those found in our study: the importance of the manner in which research is conducted, altruism (to help research) and positive benefit of participation. In their postal questionnaire for participants of the MAGPIE (Magnesium Sulphate in Preeclampsia) trial, Smyth et al. [16] report that the majority of women (58%) would definitely participate in the trial again with women citing similar reasons positive perceptions of the wider benefit of research as well as potential benefit to themselves or their baby from the therapy in the trial. Negative responses related to women’s experiences of side-effects or that the approach was pressurised or not at the right time. This is supported by Duregrov, who found women’s experience of research was positive or very positive valuing the opportunity to tell their complete story [17].

These studies show that research into sensitive areas are possible and may be well received by many parents, provided that the research is conducted in an appropriate manner. Interestingly, one such issue, the timing of approach was felt by some non-participants in MiNESS to have been too soon, appears to be different to the effect observed in the Sydney Stillbirth Study (SSS) [18], which had a high participation rate (85%). In SSS, research interviews took place within three days of the stillbirth compared to a median interval of 25 days in MiNESS, suggesting that a rapid approach had a higher participation rate. However, there were no qualitative data from SSS to assess whether this affected participants’ experiences.

The issues outlined above are not unique to research projects in pregnancy. An exploration of non-consent to research in a study of older people in which people who did not participate in the original research study were contacted again and asked why they’d declined [19]. For the majority of the people their non-participation did not reflect an objection to participation in research per se but stemmed from barriers or misunderstanding about the nature or process of the project itself. This emphasises the need for accessible, clear information for research participants especially if potential participants have no previous experience of clinical research.

Practically speaking, more information about participants’ views and experiences of participating in clinical research are needed to enhance the design of studies; this is especially important when considering emotive topics such as stillbirth. It is also important to consider the views and experiences of specific groups of women who may have lower rates of participation e.g. women from black or minority ethnic groups, to determine whether barriers and facilitators for participation are similar or whether specific issues need to be addressed.

## Conclusions

In summary, this study identified several means to facilitate recruitment: a need to ensure potential participants are well informed about research and its potential benefits to build on positive experiences and to consider the timing of the approach, and for initial information about the study to be given through a trusted caregiver. Lastly, efforts to address the stigma around stillbirth are required, as this was identified as a barrier to participation in research studies relating to stillbirth, as well as to health promotion activities and provision of care for parents in general [20]. These facilitators for requires continued collaboration between maternity, public health and third-sector organisations to maximise the number of and recruitment to studies relating to stillbirth.

## Abbreviations

MAGPIE: Magnesium Sulphate in Preeclampsia
MiNESS: The Midland and North of England Stillbirth Study
PPI: Payment Protection Insurance
SSS: Sydney Stillbirth Study
